# Cerebellum: What is in a Name? Historical Origins and First Use of This Anatomical Term

**DOI:** 10.1007/s12311-020-01133-7

**Published:** 2020-05-13

**Authors:** Jan Voogd, Chris I. De Zeeuw

**Affiliations:** 1 Department of Neuroscience, Erasmus MC, Rotterdam, The Netherlands; 2 Royal Dutch Academy of Arts and Sciences, Netherlands Institute for Neuroscience, Amsterdam, The Netherlands

**Keywords:** da Vinci, Hundt, Galen, Puppi, Cerebellum, First picture

## Abstract

In this paper, we study who first used the Latin anatomical term “*cerebellum*” for the posterior part of the brain. The suggestion that this term was introduced by Leonardo da Vinci is unlikely. Just before the start of the da Vinci era in the fifteenth century, several authors referred to the cerebellum as “*cerebri posteriorus*.” Instead, in his translation of Galen’s anatomical text *De utilitare particularum* of 1307, Nicolo da Reggio used the Latinized Greek word “*parencephalon*.” More peculiar was the Latin nautical term “*puppi*,” referring to the stern of a ship, that was applied to the *cerebellum* by Constantine the African in his translation of the Arabic *Liber regius* in the eleventh century. The first to use the term “cerebellum” appears to be Magnus Hundt in his *Anthropologia* from 1501. Like many of the anatomists of this period, he was a humanist with an interest in classical literature. They may have encountered the term “*cerebellum*” in the writings by classical authors such as Celsus, where it was used as the diminutive of “*cerebrum*” for the small brains of small animals, and, subsequently, applied the term to the posterior part of the brain. In the subsequent decades of the sixteenth century, an increasing number of pre-Vesalian authors of anatomical texts started to use the name “*cerebellum*,” initially often combined with one or more of the earlier terms, but eventually more frequently in isolation. We found that a woodcut in Dryander’s *Anatomia capitis humani* of 1536 is the first realistic picture of the cerebellum.

## Unlikely Involvement of Leonardo da Vinci

For us, the anatomical term “cerebellum” denotes the posterior part of the brain. But by whom and when was this term introduced? This question was discussed at the recent cerebellar meeting in Tokyo, dedicated to the late Professor Masao Ito. It was suggested at the time that the term was introduced by Leonardo da Vinci. Bergland [8], indeed, reported that his father, a Swedish neuroscientist, stated that da Vinci coined the term “*cerebellum*.” But did da Vinci (1452–1519) use Latin anatomical terms in his drawings? Antonio de Beatis, in his account of a visit of Cardinal Luis of Aragon to da Vinci in Amboise in 1517, states: “he has written of the nature of water, of divers machines and of other matters, which he has set down in an infinite number of volumes, all in the vulgar tongue …” (see also [45]). Moreover, there are Italian but no Latin anatomical terms in a glossary of a set of anatomical drawings of the musculoskeletal system from about 1510 [15]. For the brain, da Vinci used the terms “*celabro*,” “*cielabro*,” and “*ciervello*” (Jonathan Pevsner, personal communication). The diminutive of “*ciervello*,” “*cierveletto*,” is never used. da Vinci’s drawings of the nervous system were considered in four books [14, 41, 42, 50] and in the paper of Gross [34]. No mention is made of the cerebellum in these works, Larink even denies da Vinci’s use of the term. In the translations of the text on da Vinci’s drawings by MacCurdy [45] and Keele and Pedretti [41], there is no mention of the term “*cerebellum*” either*.*

**Fig. 1:**
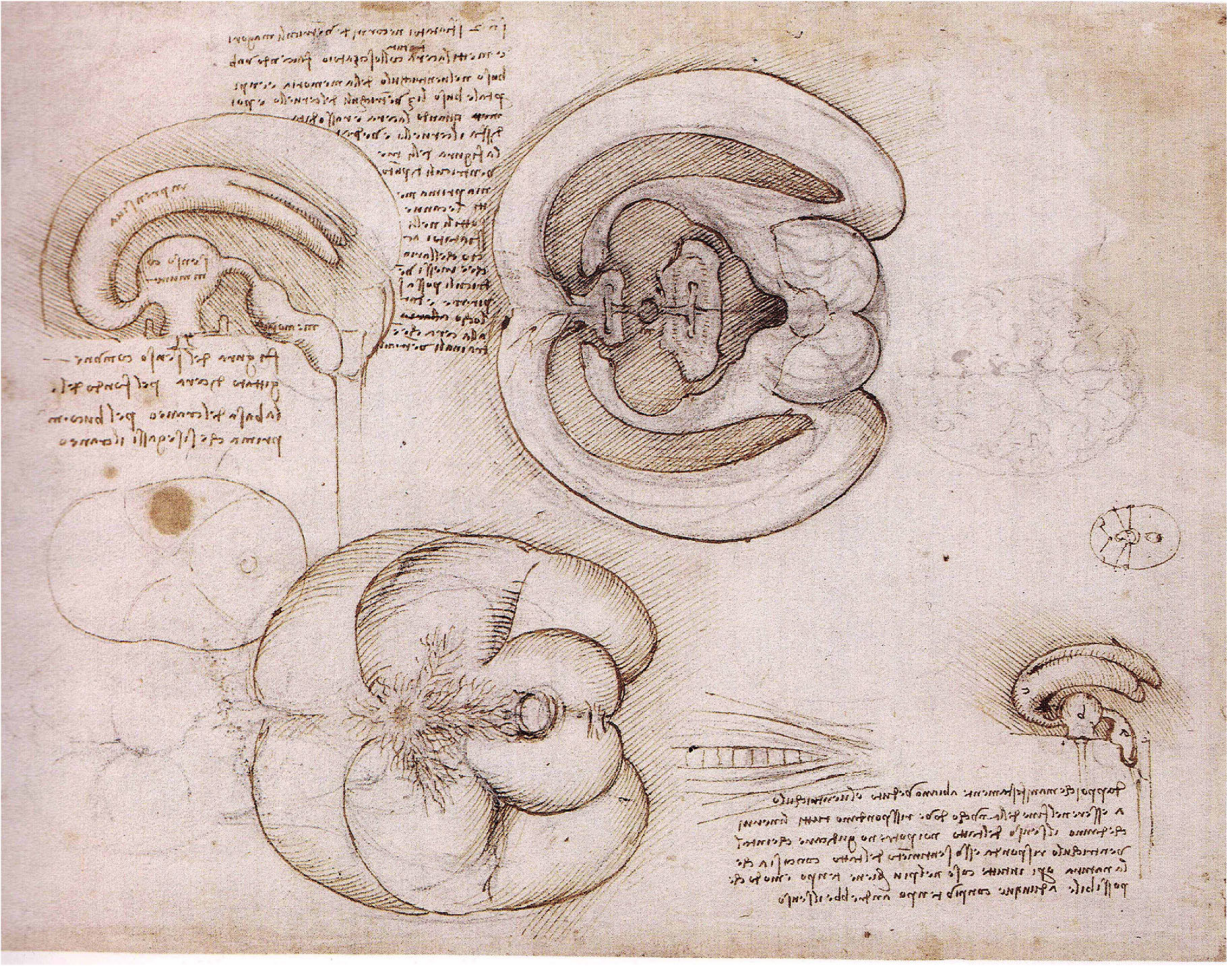
Text and drawings on this sheet of da Vinci are devoted to the preparation of molds of the ventricular system. Upper left is a lateral view of the ventricles. In the center, the ventricles are projected onto the facing halves of a sagittally sectioned brain. Lower right is an oblique view of the ventricles, with the fourth ventricle continuing in the spinal cord. Lower left a ventral view of the brain of an ox

There is only one sheet among da Vinci’s anatomical drawings that includes the contour of the cerebellum (Fig. 1). This sheet shows a sagittally sectioned brain with the outline of the ventricular system and a ventral view, presumably of an ox, showing the rete mirabile. The ventricles in the upper left are inscribed with their functions: “*impressiva*” for the lateral ventricles, “*senso commune*” for the 3rd ventricle, and “*memoria*” for the 4th. These denominations, which are repeated in two other sheets with diagrams of the ventricular system, are remarkable, because the “*senso commune*” is usually attributed to the lateral ventricles, and the 3rd ventricle is usually referred to as “*ratio*.” The text here describes the method to prepare molds of the ventricular system by injecting molten wax through holes in the “*ventriculi magori*” (the lateral ventricles). The ventricles in the lower right are indicated with “a,” “b,” and “c,” respectively. You can recognize the reversed script, because the “b” reads like a “d.” The text in the lower right corner reads: “*Dappoi che manifestamente abbiamo veduto el ventrichulo a essere nel fine della nuca, dove rispondano tutti il nervi chedanno ilsenso deltatto noi potreno gudjcare cheintal ventrichulo risponda esso al sentimeto deltatto concosia che la natura operi intutte cose nel piu brieve tempo e modo che possibile adunque con piu tempo andrebbe il senso*” (as we have clearly seen that chamber a (fourth ventricle) is located at the end of the spinal cord, where all nerves that mediate the sense of touch come together, we can conclude that the sense of touch passes through this chamber, because nature uses the shortest way for all things.) Therefore, this sense will take more time. (transcription and translation, [41]).

In his drawings of the brain, da Vinci never used specific names for its subdivisions. He mentions the “*musculo detto verme*” (the cerebellar vermis) “that is located in one of the ventricles and that lengthens and shortens, to open and close the connection between *senso commune* (third ventricle) and *memory* (the fourth).” So the vermis is seen as a valve, one of Galen’s constructs. The “two kumquat brain hemispheres,” to which Bergland [8] refers, are not visible on this or any other sheet of da Vinci.

At an age of over forty, da Vinci decided to learn Latin to have access to scientific works (Site Carnet de Léonard de Vinci; bibliothèque de l’institut de france). Different codices such as Manuscripts H of da Vinci, dating from around 1495, contain a copy of a Latin grammar by Nicoló Pérotti and a large part of a contemporary Latin vocabulary by the poet, Luigi Pulci [12]. It seems unlikely that this vocabulary contained Latin anatomical terms. According to Clayton and Philo [14] as well as MacCurdy [45], da Vinci had access to the Latin anatomical texts of Mundino, Galenus, and Albertus Magnus. Keele and Pedretti [41] mention that da Vinci owned an Italian copy of de Ketham [17] books that contained an Italian translation of Mundino’s Anatomia. However, none these texts used the term “*cerebellum*.” Leonardo may have owned a copy of Benedetti’s book “*Physici Anatomice sive Historia Corporis Humani*” of 1502. In this book, Benedetti used the term “*cerebellum*,” but this information reached da Vinci late in his life. Our conclusion is that it is unlikely that da Vinci ever used the term “*cerebellum*,” although it is not possible to completely exclude it.

## Ancient Greek, Arabic, and Early Christian Sources for the Anatomy of the Brain

**Fig. 2:**
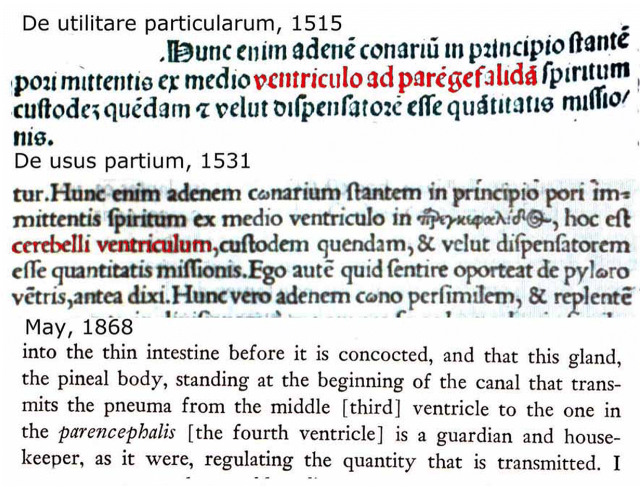
In the printed edition of *De utilitare particularum* of 1515, the term “*parencefalide*” is used for the *cerebellum*. In the edition by Simon de Colines of 1531, this is changed at some places by “cerebellum.” Translation by Tallmadge May (1968)

The main source of anatomical knowledge for pre-Vesalian anatomists of the early sixteenth century as well as the preceding middle ages was Galen. Galen (129–199 AD) was a Greek physician born in Pergamon and author of numerous medical texts. He lived in Rome and was the personal physician of several emperors. His anatomical observations are based on the dissection of animals, particularly oxen and monkeys. He never dissected a human body. One of the first Latin translations of Galen’s “*De Utilitate Particularum*” (on the use of parts of the human body) from Greek into Latin was made by Nicolao de Reggio in 1307. This translation appeared in print in the Surianus’ edition [28] and in Galen’s Opera Omnia by de Burgofranco [29]. De Reggio used the Latinized version of Aristotle’s Greek term “παρεγκεφαλίς” *parencephalon* for the cerebellum. Other Greek terms for the cerebellum, such as “e*ncranion*” or “*epencranis*” as used by the Alexandrian anatomists Herophilos and Erasistratus in the third century BC, were also mentioned in de Reggio’s translation, but “*cerebellum*” was never used. Another edition of this work was provided by Simon de Colines in Paris in 1528 [30]. This version was substantially modified with a tendency to conform to the humanistic style of the period, fitting de Reggio’s translation into a more Ciceronian mold [24]. In this edition, Simon de Colines utilized the “modern” term “*cerebellum*,” but “*parencephalon*” was still used (Fig. 2). Under the name “*De Usu Partium*,” De Colines’ edition remained one of the few translations available till the nineteenth century. Translations of Galen’s “*De Anatomicis Administrationibus*” have been provided by Demetrius Chalcondyles and edited by Berengario da Carpi as *De Anatomicis agressionibus* in the *Libri Anatomici* published in Bologna by Phaelli (1529) [31] and Guinter von Andernach (1531) [32]. This work was translated in English by Singer [59].

**Fig. 3:**
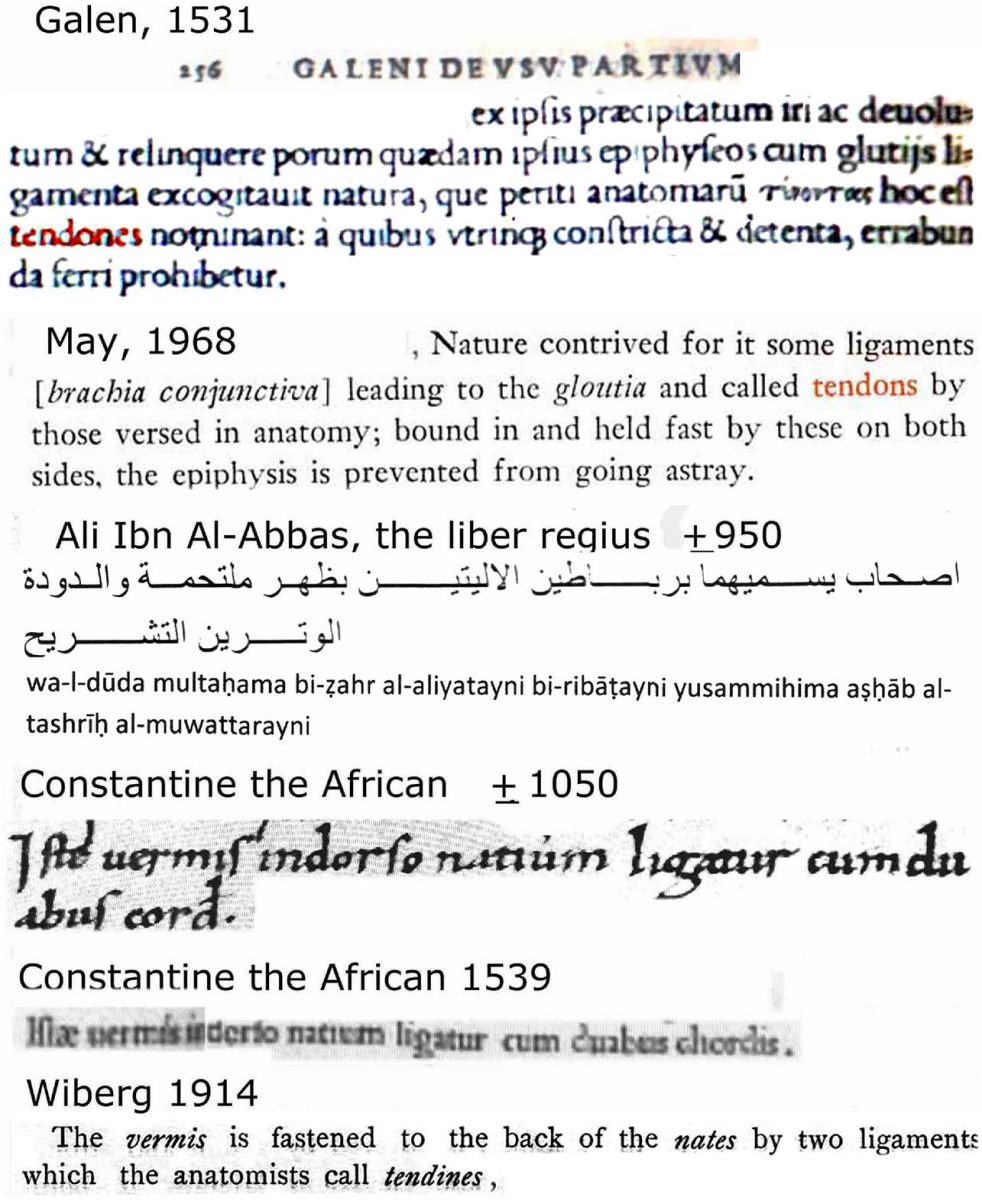
Galen (1531) describes the relief on both sides of the Velum medullae anterius, caused by the superior cerebellar peduncle, as the tendons that connect the cerebellum with the *glutaei* (the buttocks, our present lamina quadrigemina). This statement is copied in Ali Ibn Al-‘Abbas Liber regius. Constantine the African translates this sentence in the 1050 manuscript and the 1539 printed edition of the *Liber pantegni*. Translations by Tallmadge May (1968) and Wiberg [68]

Galen’s anatomy is often discarded as a kind of veterinary anatomy, misleading for students of the human body. But his descriptions are full of detail and have not been improved upon till the appearance of Fabrica (1543) by Vesalius [66], who broke with Galen’s tradition by using dissection of the human body as his main source of anatomical knowledge. In “*De Usu Partium*,” Galen recognized the difference in appearance of the parencephalon and the cerebrum. “It (*the cerebellum*) is composed not of large convolutions separated by the thin membrane (the pia mater) like the encephalon (the cerebral hemispheres), but of many small bodies differently arranged from those of the encephalon” (translation by Tallmadge May, 1968). In another paragraph, Galen described the superior cerebellar peduncles, bordering the velum medullare anterius that contains the lingula, the ventralmost lobule of the cerebellum [67]. “Since for all reasons it (the lingula of the cerebellar vermis) would tend to be easily moved in various ways, there was danger that as it was carried upon the convex back of the *gloutia* (the buttocks, the lamina quadrigemina) and roll off to one side and abandon the canal (the anterior fourth ventricle). Nature contrived for some ligaments leading to the *gloutia* and called tendons (our superior cerebellar peduncles) by those versed in anatomy; bound in and held fast by these on both sides, the *epiphysis* (Galen’s term for the cerebellar vermis) is prevented from going astray” (Fig. 3). Galen considered the vermis as a valve regulating the flow of animal pneuma between the third and the fourth ventricles. One of Galen’s preoccupations was with the texture of the forebrain and the cerebellum. The forebrain is soft and gives rise to the soft, sensory cranial nerves. The optic nerve and the tractus olfactorius develop as evaginations of the brain and, indeed, are fragile. The hardness of the cerebellum is impossible to check, but the nerves that originate from the cerebellum (he probably meant from the brainstem, but this structure was not recognized by Galen) are mostly motor nerves and hard by the perineurium that covers them. Perhaps we can better appreciate Galen’s work today, because most of our present knowledge of structure and function of the brain is based on animal experiments. The revival of human neuroanatomy with the introduction of computed axial tomography and magnetic resonance imaging is only a recent event.

Galen’s teachings also became known, indirectly, through Syriac and Arabic translations of his works. One of the translators of Galen was Hunayn Ibn Ishan, Johannitius by his Latin name (807–877), a Nestorian Christian. His translations, presumably, were used in the *Kamil as-sinaa at tibbya* (the perfect book of the art of medicine) or the *Kitab al-Mliki* (the Liber regius) by Ali Ibn al-‘Abbas al Majusi (930–994), an important medical text [18]. It was translated into Latin by Constantine the African as the *Liber Pantegni*, the first comprehensive medical text in Latin [40] and the earliest treatise on anatomy of the early middle ages [16]. Constantine (1010–1091) was born in North Africa, traveled around to collect Arabic manuscripts and moved to Italy where he became a Benedict monk in the Abbey of Monte Cassino and taught at the medical school of Salerno, the Collegium Hippocraticum. Encouraged by Robert Guissard, the Norman Duke of Apulia, he translated several Arabic manuscripts into Latin. His contemporaries accused him of plagiarism because he never mentioned the Arab authors of the texts he translated and thus made the impression that he conceived these texts himself. In the twelfth century, Stephen of Antioch again translated the book because he was dismissive of Constantine’s Latin text. The oldest manuscript of the Liber Pantegni, dating from the eleventh century and possibly written and supervised by Constantine himself in the Monte Casino, is present in the Royal Library in The Hague. The first printed version of the Pantegni dates from 1539 [2]. Both versions are available on the internet. The Pantegni is divided into theoretical and practical parts, each of them subdivided into ten books. Chapter 11 of book 3 of the Theory is concerned with the anatomy of the brain [35]. It was translated into English by Wiberg [68].

**Fig. 4:**
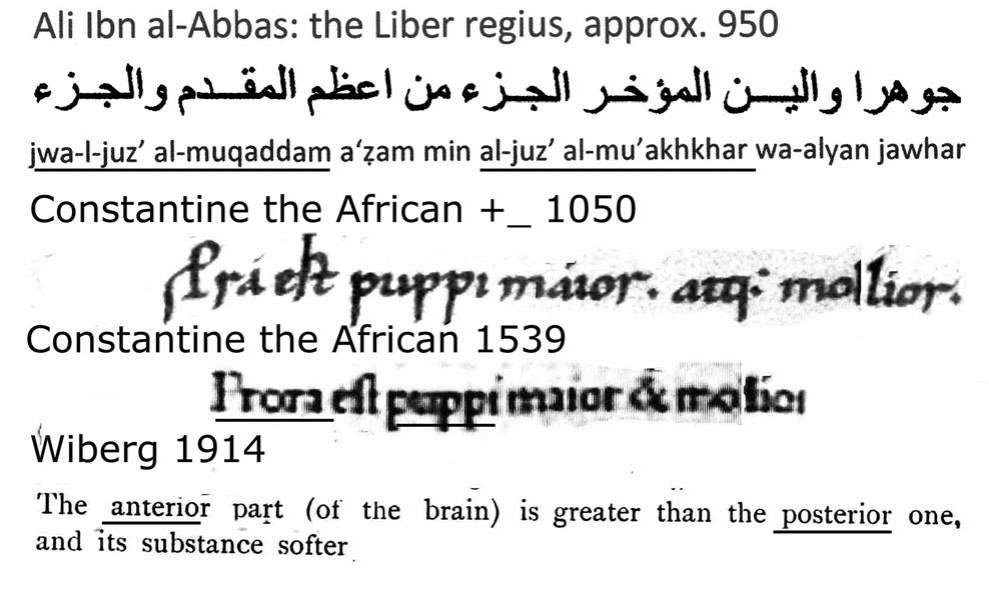
Arabic text of Ali Ibn al-‘Abbas’ *Liber regius* and its transcription. The 1050 manuscript and the printed edition of 1539 of its translation by Constantine the African as the *Liber Pantegni*. In the 1050 manuscript and the printed edition of 1539 the sentence: “Prora (anterior part of the brain) est puppi (posterior, cerebellar, part of the brain) major & mollior,” “*prora*” is abbreviated in the manuscript. Anterior and posterior and their Arabic and Latin equivalents are underlined. Translation, Wiberg [68]

Bos [9] noticed that Constantine the African, in the translation of another text, the *Liber de oblivione*, translated the Arabic terms for the anterior and posterior parts of the brain as the Latin “*prora*” and “*puppi*,” respectively. In the *Liber pantegni*, his translation in Latin of the *Liber regius* of Ali Ibn al-‘Abbas, he used the same terms as in the sentence of Fig. 4: “*Prora est puppi major ata mollior*” (the anterior part of the brain is larger and softer than the posterior part). Constantine and his colleagues had to find new Latin words for new medical subjects. He introduced the nautical terms “*puppi*” and “prora,” the stern and the bow of a ship, to indicate the cerebellum and forebrain, respectively. The French “poupe” and the English “poop” are derived from the Latin “puppi.” The possibility that Ali Ibn Al-‘Abbas’ Liber Regius was based on Galen’s texts is suggested by the paragraph on the tendons (the superior cerebellar peduncles), which appears like a perfect copy (Fig. 3).

A hundred years later, the medical treatises of Muhammed Ibn Zakariyya Al-Razi (Rhazes) from the ninth century [10] and Abu’ak Husain ibn Abdullah ibn Sina (Avicenna) dating from the eleventh century were translated in Toledo from Arabic into Latin. The anatomical text of Rhazes “*Al mansuri*” does not provide anatomical information on the brain. Chapters in Avicenna’s Canon, translated in French by de Koning [18], reiterate Galen on the topographical subdivisions of the brain and the ventricles, on the texture of the fore- and hindbrain in relation to the sensory and motor nerves, and on the tendons (the superior cerebellar peduncles) connecting the cerebellum with the glutaea (the tectum).

One of the church fathers, Nemesius, the Byzantine bishop of Emesa (the present Homs), wrote the tract “On the nature of man” [64] sometime before the year 400, addressing the relation between soul and body. The brain and its nerves are discussed as the bearers of mental attributes. The Greek text was translated into Latin by Alfanus of Salerno, a contemporary of Constantine the African in the Abbey of Monte Cassino. A much improved translation was made in the twelfth century by Burgundio of Pisa. Burgundi was a judge, engaged in negotiations between Rome and Byzantium. Nemesius is one of the first to discuss the functions of the three ventricles: “*Organum autem et huiu est et posterior ventriculus cerebri, quem παρεγκεφαλίς et παρεγκρανϊ vocant, et qui in eo est animalis spiritus. Quia vero sensum quidum principia et radices anteriores esse ventres diximus cerebri, discretivi vero eum est medius, memorativi vero posteriorem, necesse est demonstraresi haec hoc modo habent, …*” (“The organ of this faculty (the faculty of memory) is the hind part of the brain also called “*parencephalo*” and “*pericranis*” and the vital soirit there contained. Now if we make the assertions that the senses have their sources and roots in the front ventricles of the brain, that those of the faculty of intellect are in the middle part of the brain, and that those of the faculty of memory are in the hind part of the brain, we are bound to demonstrate that this is how these things work, …” [67]). Nemesius retains Galen’s observations on the texture of the cranial nerves: “*Emittuntur autem qui sensibilis quidem et molles nervi a medio et anterioribus ventriculis cerebri; durioris vero et motivi a posteriore ventriculo et spinali medulla*” (“The soft nerves of sensation - i.e., the optic nerve and the tractus olfactorius - descend from the middle part and from the front lobes of the brain, while the harder motor nerves proceed from the posterior lobe and the marrow of the spine.” (translation [61])). The term “*pericranis*” for the cerebellum is not found elsewhere in the literature and may be a term current among the Byzantine physicians of Nemesius time.

## The Term Cerebellum Was Not Used for the Posterior Part of the Brain Before 1500

Three Latin texts from the first half of the twelfth century are based on lecture notes taken at public dissections of a pig at the Salerno medical school. The *Anatomia Cophonis* is a fairly short tract. It was later included by Dryander in his book in 1537. *The anatomia Mauri* is very similar [53]. Neither of them contains a description of the brain. The *second Salernian demonstration* contains a cursory description of the brain: “It (the head) is tapering in front, because of the chamber of imagination and the sensory nerves, which proceed to the organs of sensation, and it tapers behind, because of the chamber of memory and the motor nerves, which run to the organs of locomotion and because the spinal medulla makes its exit at the rear,” (translation [16]). According to this author, these texts are mainly based don Constantine’s *Liber pantegni*. However, they do not use Constantine’s terminology for the fore- and hindbrain.

A hundred years after the appearance of the *Pantegni*, the twelfth century anatomy from the *Codex Latinus* [54] still uses Constantine the African’s terms for the fore- and hindbrain: “*Sividur caput itaque secundum unam divisionum in proram et puppi et secundum hoc prora apellatur anterior pars capitis ab en loco, quo memorialis cellula*- a mistake: should read “fantastica” *-* cellula conjungitur reliquae partis capitis, Puppis apellatur memorialis cellulla.” (As we divide the head in the forebrain and the hindbrain, we nominate prora as the anterior part of the head, and locate the cellula of the phantasy in this part of the head. Puppis is the name for the cellula of the memory). Two very similar thirteenth century manuscripts, “*Anatomia Ricardi Salamitani*” and “*Anatomia magistri Nicolai physici*” [54] indicate the ventricles after their function as in “*nervi omnes motivi orientur a memoriali cellula, quem as modem sensibilis a phantastica*” (“motor nerves originate from the fourth – memory - ventricle, sensory nerves from the lateral – phantasy - ventricles”) [16, 54].

(Saint) Albertus Magnus was a German Dominican monk who wrote a commentary on Aristotle’s “*de animalibus*” (1268) under the same name [3], a text that was part of the medical curriculum, because of its anatomical and general biological information. The first chapters of Albertus Magnus’ text “*de animalibus*” are concerned with human anatomy. He distinguished the three ventricles on the basis of their function (*distinctiones cellularum capitis ad imaginandum*, *estimandum*, *memorandum*) and explains Constantine the African’s terminology (“*anterius prora et posterius puppi*s”) in his description of the head. Sensory nerves are moist and soft and originate from the front, while voluntary motor nerves (“*nervorum motivorum*”) are dry and hard and originate from the rear. In his account of the texture of the brain, he indeed uses topographical terms (“Substantia cerebri … queniam anterior pars eins est mollior et lenior et posterior siccior et durior”) (of the brain the anterior part is moist and mild, the posterior dry and hard). He reports the longitudinal division of the brain in two, right and left, parts and notices that the posterior (cerebellar) part of the brain is less complex (“Et in posteriore parte capitis est cerebrum aliud secundum complexionem”).

A few years earlier, Albert Magnus (or rather his student Conrad of Austria), published *Quaestiones super de animalibus* [4], a text reporting Albert’s lectures on Aristotle in Cologne. These lectures consist of a series of questions and answers. The answers usually comprise a thesis and an antithesis. One remarkable question reads: “Why the brain is divided into front and rear and not right and left,” with the answer: “Since other members are divided into right and left, like the eye and ear, the hand, foot etc. Therefore the same scheme should apply to the brain. To this argument one must reply that sense and motion arise in the brain. But the senses, like sight, smell and taste, flow from the anterior part of the brain, and this is why the posterior part of the brain is neccessarily left for the motive powers. And that is the reason the brain is divided into two parts of which the first is moister owing to the influence of sensitive powers, while the posterior part is dryer owing to the motive powers” (translation [56]).

The *Anatomia vivarum* (*Anatomia Ricardi anglici*), a text dating from the early thirteenth century, originally was attributed to Galen. In the text, topographical terms are used to indicate the subdivisions of the brain: “*Item cerebrun in anteriorei sui parte mollius est et humididius propter nervos sensibilis inde orientes qui molliores habent esse quam nervi motini. In posteriori sui parte durius est propter nervos motios*” [63] (“The brain is softer and moister in its anterior part on account of the sensory nerves arising there, which have to be softer than the motor nerves. In the posterior part it is firmer on account of the motor nerves arising there”) (translation [16]). The text is based on the Latin translation of Arabic texts of Avcenna that date from the late twelfth century. According to Redeker [54] “the lowest point in the decline of the barbaric Latin of the monks” is reached, with the Latin of the *Anatomia vivarum*.

Surgeons in the early middle ages often wrote texts on their profession. These “surgeries” usually contained sections on the anatomy. William de Salicete (1210–1285) got his medical education in Bologna, practiced surgery in several Italian cities, and published on anatomy in his *Chirurgia in summa conservationis et curationis* of 1275. Book four of his opus contains a short description of the brain “Et (cerebrum) dividur totaliter partes scilicet anteriorem, medium et posteriorem.” The term “*pars posteriorem*” for the cerebellum occurs once again in the sentence “Sub capite, in parte posteriori est nucha” (under the head, in the posterior part of the brain is the spinal cord) (Translation [37]).

The well-known French surgeon Henri de Mondeville published his Chirurgie around 1300 in a Latin [20] and a contemporary French edition [21]. It describes the subdivision of the brain as the ventricular system as follows: “*La pie mere touche le cervel sans moien et la devise apparissablement en 3 ventres … la quelle devision apert plus profonde ou ventrail devant, de telle manière qu‘il semple etre double … et est comprise en lui la vertu imaginative et fondee, la quelle reçoit du sens commun les especes des choses sensibles de hors … Le ventrail du millieu est plus petit que les autres, ou quel et la vertu resonable … Puis est la 3 ventrail, ou quel est fondee la vertu memorative … Et de la partie derriere de cestui 3 ventrail pars dessus en est la nuche* …” (“The pia mater touches the brain and divides it in three ventricles. This division is more extensive in the front where the ventricle seems to be divided in two parts. The faculty of the imagination is located in this ventricle, which receives the common sense as sensory impressions from the surrounding World. … The middle ventricle is smaller than the others, it locates the faculty of reason … Next is the third ventricle, where the faculty of memory is located … Behind this third ventricle the spinal cord is located …”)

**Fig. 5:**
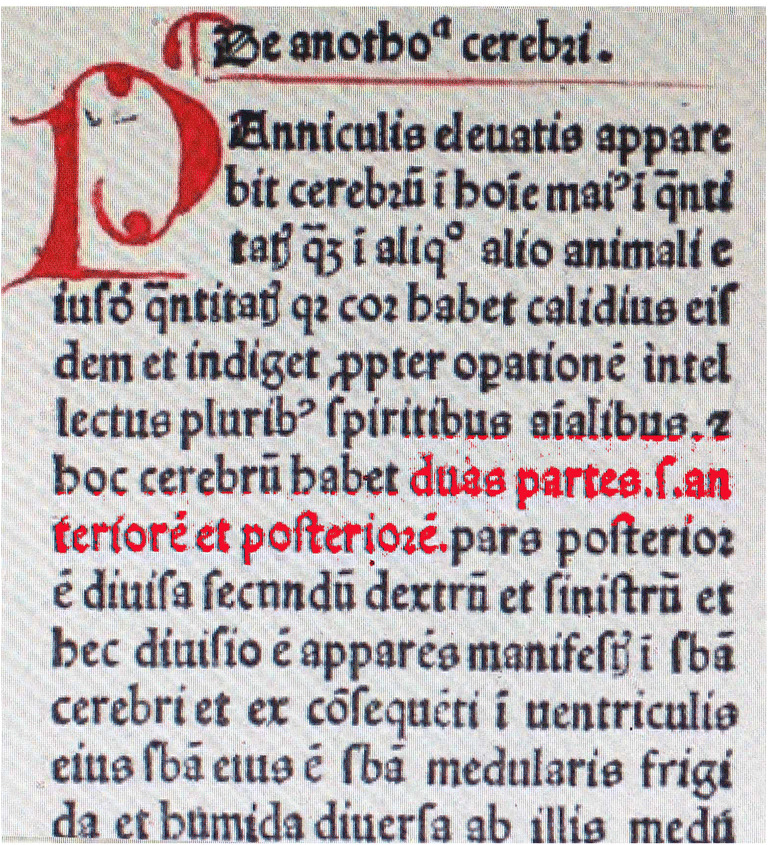
In Mundinus Anatomia (1487) the terms “*cerebri anterius*” and “*posterius*” are used for the cerebrum and cerebellum, respectively. “When the membranes have been seen, the brain will appear larger in quantity in man than in any other animal of the same size, because he requires more animal spirit for the operation of his intellect. The brain has two parts, anterior and posterior, and the anterior part is divided into right and left; this division appears clearly in the substance of the brain and consequently in the ventricles.” Translation, [13]

Mundino de’ Luzzi was a contemporary of de Mondeville in Bologna. His *Anathomia* [47] (Fig. 5), a dissection guide and a popular anatomical text, dates from around 1310; it was first printed in 1487 [47]. Like his contemporaries, he used the term “*cerebri posterius*” for the cerebellum [13]. In the Italian manuscript of the anatomy of the Bolognese surgeon Hieronymus Manfredi (1430–1493) [58], dedicated to the ruler of Bologna, Giovanni Bentivoglio, Mundino’s nomenclature was used: “*Questi dui pannicuili in piu luochi penetrano la substantia del cerebro et se divide in parta drita, e parta sinistra et in parta anteriore & parte posteriore*” (Two meninges penetrate in the brain and divide it into a right part and a left part as well as an anterior part and a posterior part). The same terms for the forebrain and the cerebellum are used in Guy de Chauliac’s *Chirurgia magna* of 1363 [62] and in Johannis de Ketham’s *Fasciculos di medicinae* (1491): “Et ideo dividit anteri cerebri a posterior.”

Johann Peyligk was a council member of the Faculty of Arts of the University of Leipzig. The physical chapter of his *Compendium*: *Philosophiae naturalis* of 1490 [51] was published again in 1513 as the anatomical text *Compendium Capitis Physici Declaratio Principatium Humani Corporis* [52]. It opens with an “Inscription to the beholder”:

Would’st know the human body’s every part

Its uses and its properties by book?

Each in its natural form described by art

Here shalt thou clearly find. Beholder, look!

(Translation [43]).

Unfortunately, “every part” does not include the cerebellum. According to Lind [44], the book represents the backward state of anatomy in German countries. The book is one of the first to use illustrations of the anatomy.

## Rise of the Term “Cerebellum”

**Fig. 6:**
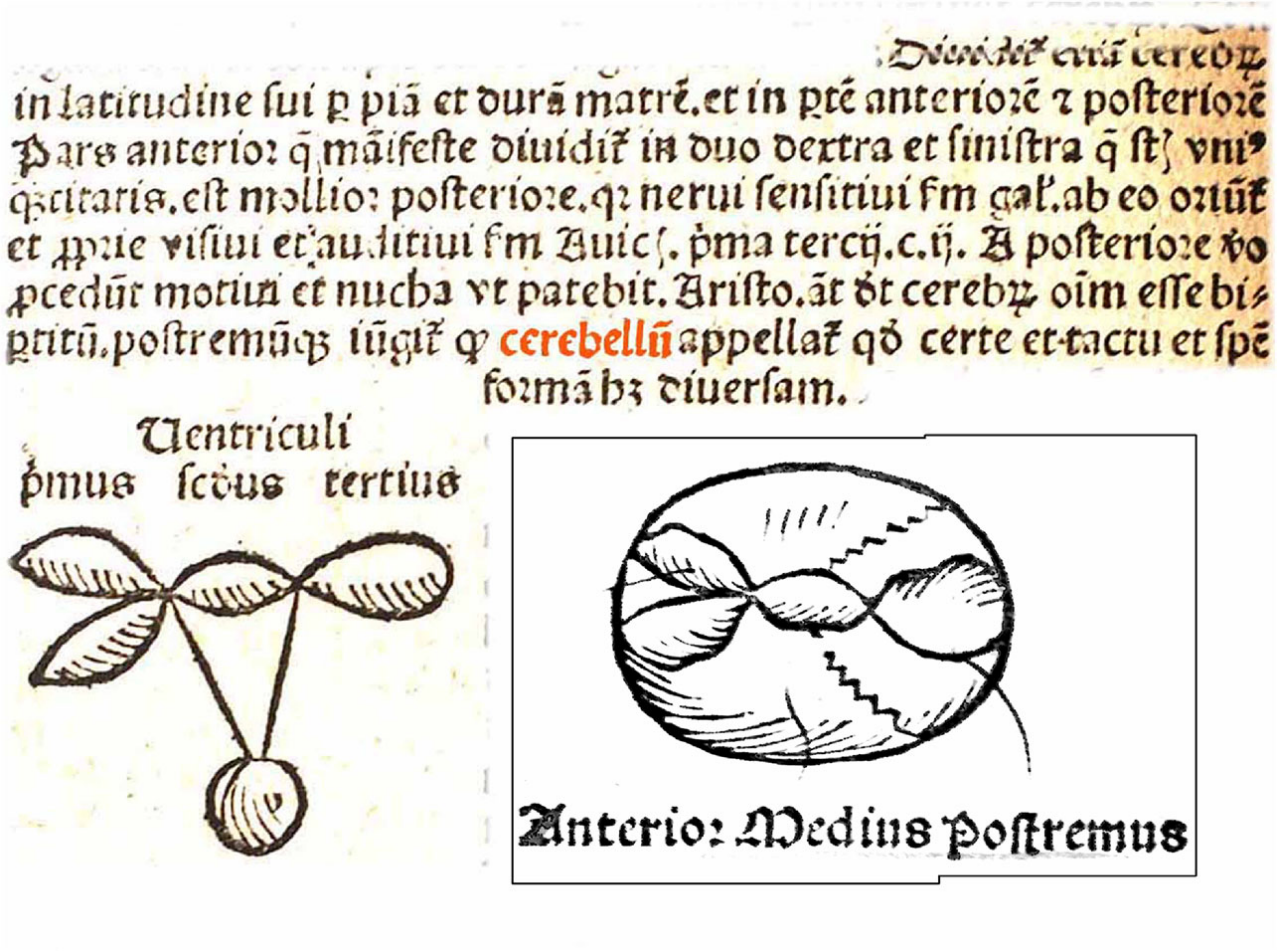
Hundt in his *Athropologium* from 1501 is the first author of an anatomical text to use the term “cerebellum” in the modern sense. “Dura and pia divide the brain in anterior and posterior and right and left parts. The anterior part is softer than the posterior part, sensory nerves take their origin from the former. The third ventricle gives origin to the motor nerves and spinal cord. The posterior division is also called cerebellum.” The diagram illustrates the ventricular system. This figure is reproduced from Peyligk [51]. Peyligk uses topographical terms to indicate the ventricles. Hundt is one of the first to use numbers for the different ventricles. The first ventricle is double. The second ventricle gives rise to the infundibulum with the hypophysis

The one who was the first to use the term “*cerebellum*” for the posterior division of the brain, probably, was Magnus Hundt. In his *Anthropologia* [38] (also a new term) from 1501, he used the term “*cerebellum*” next to “*cerebri posterior*.” As “*cerebelli appellar*,” it appears for the first time in an anatomical text (Fig. 6). Hundt worked at the same University as Peyligk. The diagram of the ventricles in Fig. 6 is copied from Peyligk’s book.

**Fig. 7:**
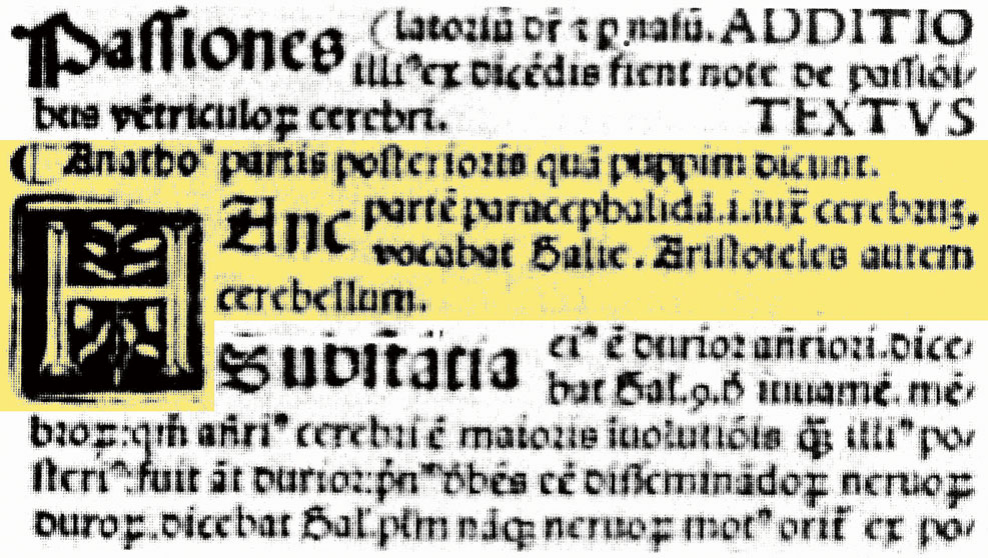
Zerbi in his book *Liber anathomie* from 1502 uses Constantine the African’s term “*puppi*” for the cerebellum, but also mentions “*parencephalon*” and “*cerebellum*” as synonyms

In the following decades of the early sixteenth century, the term “*cerebellum*” became known to most pre-Vesalian anatomists. Gabriel Zerbi in his *Liber anatomie* from 1502 used, apart from “*puppi*,” “*partes posterioris*,” and “*parencephalon*,” also the term “*cerebellum.*” For example, when he writes “*Anatho*’ *partis posterioris qui puppim dicunt. Hunc parté parencephali I unë cerebrum vocabat Salic. Aristoteles autem cerebellum*” that we translate as “Anatomists call the posterior part puppi. This part of the brain is called parencephalon by De Saliceto. Aristotle, however, calls it cerebellum” (Fig. 7). The references to de Saliceto and Aristotle are mistaken of course. De Saliceto uses “*parte posteriori*” for the cerebellum, and the Greek Aristotle could never have used the Latin term “*cerebellum*.” Zerbi is considered as a conservative scientist, using scholastic categories like “textus” and “additio” in his anatomical descriptions, aiming at completeness in citing from Arabic sources, such as the Pantegni with its term “*puppi*” for the cerebellum.

Apart from Zerbi, Gregor Reisch, a Carthusian monk, prior of the Klein Basel and friend of Erasmus, appears to be the only author who still used the terms “*puppi*” and “*prora*” for the cerebellum and the forebrain. We quote from his encyclopedia *Margarita Philosophica* of 1503 [55]: “*A prima oriuntur nervi sunt infiru mera mores volutarii: & ad capitis puppim prenduné. A secundà aut nervi procedent: qui per capitis prora ad singula sensuum organa … dunt de quibus supra*”; “The posterior part of the head (the cerebellum) gives rise to the voluntary nerves. Moreover, nerves proceed from the frontal part of the head to each of the sensory organs.”

Alessandro Benedetti was a contemporary of Zerbi in Bologna. Benedetti’s *Physici Anatomice sive Historia Corporis *[4] was published in the same year [69] as Zerbi’s book. Benedetti was a modern scientist, abandoned the medieval writers and, in the spirit of the humanists, returned to the Greek ground texts of Galen [26, 44]. In his book, he systematically used the Latin term “*cerebellum*.”

Allessando Achillini, who worked in Padua and Bologna, did not use the term “*cerebellum*” in his book *Annotationes Anatomicae* of 1520: “The brain is large, divided in anterior and posterior parts; one ventricle in the posterior part of the brain and the posterior brain is pyramidal in shape, because the ventricle located within it is pyramidal” (translation [44]).

Berengario da Carpi published in 1521 a 427-page commentary on Mundino’s *Anathomia* [6]. Because this book did not sell well, he published a shorter version, the *Isagogue* [7] from 1522 that became more popular. Both books use “*cerebellum*” as an alternative to Mundino’s “*cerebri posterior*” (“*cerebelli sue cerebri posterius*”). da Carpi’s *Isagogue* was indicated as the source for the term “*cerebellum*” by Swanson [60], but he is preceded by Hundt, Zerbi, Benedetti, and da Carpi’s commentary.

Guinter von Andernach, a physician, was one of the foremost scholars of antiquity and of anatomy of his time. He worked in Paris between 1527 and 1538, where Andreas Vesalius was one of his assistants and dissectors. Andernach was a contemporary in Paris of Jacobus Sylvius, whose unyielding devotion to Galen’s teachings made him one of the most outspoken criticasters of Vesalius. Guinter’s translation of Galen’s *De Anatomicis Administrationibus* [32] was published in 1531. Here he systematically started to employ the term “*cerebellum*.” In his anatomy book, “*the Institutionum anatomicarum secundum Galeni*” [36] of 1536, Guinter used the term “*cerebellum*” in the following passage: “When the skull has been cut round, it is removed … Immediately two membranes, which they call the meninges, come into view. But before you pass to examining them, note that the brain is divided into a left and right part, as well as a third part, which adjoins them at the rear; hence the Greeks call it *parenkefalis*, “beside the encephalon” and the Latins the *cerebellum* (little brain)” (translation [48]). As a protestant, Guinter left Paris for the persecutions of his fellow believers and settled in Metz and later in Strasbourg, where he did not publish on anatomy any more.

The first time Vesalius used the term *cerebellum*” was probably in a Latin note on Guinter von Andernach’s *Institutiones* (see [48]): “*quae cerebellum contegit*.” In his revised edition of Guinter’s book (1538) [65] and in the Fabrica (1543) [66], Vesalius consistently used the term “*cerebellum*.”

**Fig. 8:**
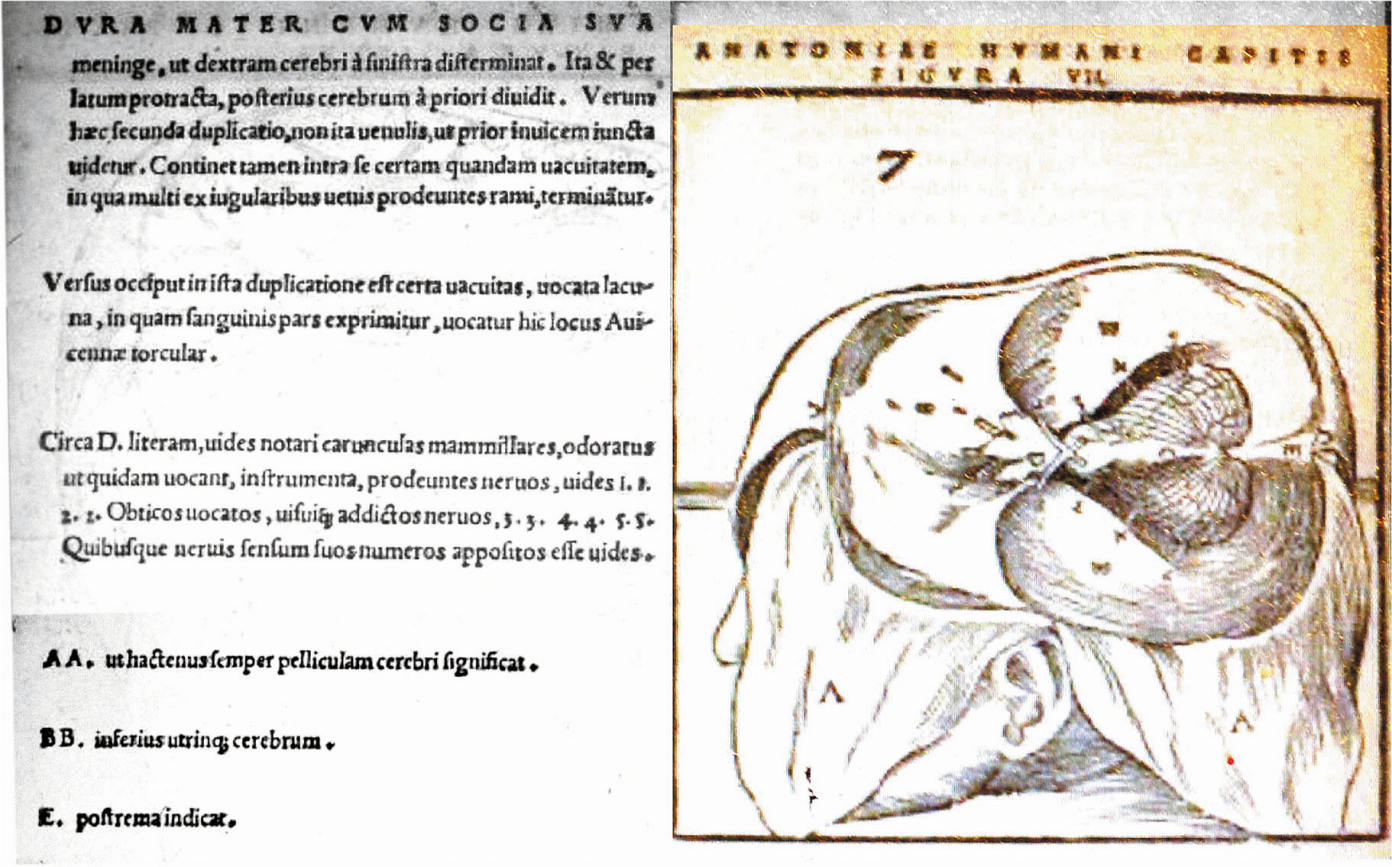
This figure presents a woodcut from Dryander’s book *Anatomia capitis humani*. It shows the stage of Dryander’s dissection of the head, where the cerebellum is illustrated as viewed through the opening in the tentorium. The legend of this figure was translated by Lind [44]. “Just as the dura mater with its accompanying meninge separates the right-hand part of the brain from the left, so also does it divide the posterior brain from the anterior. But this second duplication does not seem to be joined together with veinlets as the first one. It contains within itself, a certain vacuity in which terminate many branches of the jugular veins. Toward the occiput in this duplication is a certain vacuity called the lacuna in which part of the blood is pressed out. This is called the torcular of Avicenna. Around the letter D you see that the mammilllary carunculae are indicated (i.e., the olfactory bulbs: not visible in the illustration), the instruments of smell as some call them. They send four nerves. AA. As allways, thus far indicates the little skin of the brain. BB. The lower brain on both sides. E. indicates the posterior”

Johannes Dryander, a German scientist from Marburg, published a booklet (28 pages) in 1536, *Anatomia Capitis Humani in Marpurgensi Academiqa superiori anno publice exhibita* [22], with woodcuts by Georg Thomas of successive stages in the dissection of the head with explanatory notes, but without a detailed text, an early example of tomography. One of his woodcuts appears to be the first figure in history that clearly depicts the cerebellum (Fig. 8). In his *Anatomiae hoc est Corporis Humani Dissectiones pars prio*r of 1537 [23], an extended series of woodcuts is included with the twelfth century Salernian *Anatomia Cophonis* on the anatomy of the pig, and Zerbi’s *Infantis Clarissisima Anatomica*. In the legend of one of the figures, Dryander explains the nomenclature of the cerebellum as follows: “*Dividitur cerebrum, in anterius and posterius in dextrum and sinistrum á dura meningae secatur, ut tres cerebri partes numerentur. Posterius παρεγκεφαλίς Graeci nominant, Latini paruitate cerebellum occipir duntarat occupat* …” (“the brain is divided in anterior and posterior and in right and left parts. The dura subdivides the brain in three parts. The posterior part is called parencephalon in Greek, in Latin it is called the small cerebellum that occupies the occipital region.”)

Andres de Laguna, a Spanish scientist, published *Anatomica methodus, seu de sectione Humani Corporis Contemplatio* in 1535. He uses “posterior part of the brain” and “*cerebellum*” as synonyms. The book offers original ideas on the function of the cerebellum, as in the following passage: “People in whom this posterior part of the brain is at once rather moist and soft are talented for they are skillfull and quick to conceive or imagine, but their memory is poor; on account of their great humidity and softnes of their brains the images of things easily slip away from them. But people in which this part of the brain is harder than what is reasonable (as happens principally among the ignorant) follow some perceptions of their mind somewhat laboriously and thus are quite tenacious and preserve knowledge once they have acquired it” (translation [44]).

Niccoló Massa in his *Liber Introductorius Anatomicae* of 1536 [46] also quotes the term “*cerebellum*” (“ïntermedi cerebri anterioris & posterioris quad cerebelli apellant,” “that is called *cerebellum*”). As a physician, he published on different medical subjects. He is one of the firsts to recognize that the ventricles are filled with a fluid.

Charles Estienne was a contemporary of Guinter von Andernach and Vesalius in Paris. His book, *De Dissectione Partium Corporis Humani libri tres* [25], appeared only in 1545. It is known for its careful illustrations. He consistently used the term “*cerebellum*.”

Most of these authors of the early sixteenth century were not just anatomists but primary humanists with an interest in classical authors and active as translators of their work. Hundt was a rector and a professor of theology at the University of Leipzig. In his book, the *Anthropologia*, anatomy is just one of the chapters. Zerbi taught philosophy in Padua and medicine and logic in Bologna, till his death in 1515. He was murdered by the sons of an Ottoman sultan, who suspected him of poisoning their father. Benedetti was a professor of anatomy in Padua from 1490 till his death in 1512. He widely traveled in Greece and spoke and wrote the language. He supervised editions of classical authors such as the Natural History of Plinius. Achillini was a teacher of philosophy and medicine and published books on Aristotle. da Carpi published the Latin translation of Galen’s Anatomical procedures in 1528 with the Greek scholar Demetrius Chalcocondyles [31]. Guinter’s command of Latin was remarkable. He tried to equal the classical Latin of Celsus *De Medicina*. He translated numerous works of Galen, including the *Anatomicis Administrationibus* (1531) [32]. This translation remained the standard until the nineteenth century. Vesalius was involved in the translation and editing of several of Galen’s texts, exemplified by the frequent citation of his name in Galen’s collected works [33]. Dryander may have been an exception. He was most interested in mathematics and astronomy. His innovation was using illustrations, apart from introducing modern anatomical terms like “*cerebellum*” in his books.

## Historical Use of the Word Cerebellum as a Diminutive for the Brains of Small Animals

It is not possible to indicate a single person or source responsible for the first introduction of the term “*cerebellum*.” From the development of the different anatomical texts of Guinter von Andernach and Vesalius, which are well documented [49], it seems possible that the classical text *De Medicina* from Celsus (second century, AD) was the source [11]. Celsus uses “*cerebellum*,” the diminutive of “*cerebrum*,” for the brains of small animals. It reads: “*Deindre ex eodem sue ungulae, rostraum, aures, cerebellum, ex agno haedove cum petiolis totum caput al quanto quam cetere membra le viora sunt, adeo ut in media materia pont possint*” (which can be translated as “Then likewise in the same pig, feet, chops, ears or brain, in a lamb or kid the whole head, also the small feet are less nutritious than other parts and so can be placed in the middle class”). For Guinter, Celsus *De Medicina* was his classic example in writing Latin. In Guinter’s *Institutiones*, he justifies the use of the Latin term as follows: *cerebellum* (little brain) on account of its small size.” According to Ivanova and Holomanova [39], Celsus was also a source for Vesalius for the use of anatomical terms. Celsus is indeed mentioned in Vesalius’ *annotations* for the 1555 edition of the Fabrica [48]. Other classical authors, such as Plinius the elder, Scribonius largus, and Petronius also used “*cerebellum*” as a diminutive in their prescriptions and recipes [57]. It seems likely that authors of anatomical texts in the early sixteenth century, starting with Hundt in 1501, became aware of the use of the term “*cerebellum*” by these classical authors. As humanists, their interest in classical philology went far beyond anatomical nomenclature. Apparently, authors of anatomical texts from before 1500, who generally used the term “*cerebrum*,” were not aware of its diminutive. Albert Magnus in his *Questiones* wrote “animals with small brains” as “*animalia parvi cerebri*.” The use of the term *cerebellum* in the modern sense may have been the subject of discussions and correspondence between them, and thus its use may have become common in the early sixteenth century. However, the study of the introduction in the humanist community of this period of new Latin terms, or the application of older terms, such as “*cerebellum*,” to new concepts should be left to experts and is far beyond the expertise of the present authors.
